# Chronobiology, sleep-related risk factors and light therapy in perinatal depression: the “Life-ON” project

**DOI:** 10.1186/s12888-016-1086-0

**Published:** 2016-11-04

**Authors:** Simone Baiardi, Fabio Cirignotta, Alessandro Cicolin, Corrado Garbazza, Armando D’Agostino, Orsola Gambini, Alessandra Giordano, Mariapaola Canevini, Elena Zambrelli, Anna Maria Marconi, Susanna Mondini, Stefan Borgwardt, Christian Cajochen, Nicola Rizzo, Mauro Manconi

**Affiliations:** 1 Department of Head, Neck and Sensory System, Neurology Unit, Sant’Orsola-Malpighi Hospital, University of Bologna, Bologna, Italy; 2 Sleep Medicine Center, Neuroscience Department, AOU Città della Salute e della Scienza - Molinette, Università di Torino, Torino, Italy; 3 Sleep and Epilepsy Center, Neurocenter of Southern Switzerland, Civic Hospital, Lugano, Switzerland; 4 Centre for Chronobiology, Psychiatric Hospital of the University of Basel, Basel, Switzerland; 5 Transfaculty Research Platform Molecular and Cognitive Neurosciences, University of Basel, Basel, Switzerland; 6 Department of Health Sciences, Università degli Studi di Milano, Milan, Italy; 7 Sleep Medicine Center, San Paolo Hospital, Milan, Italy; 8 Department of Obstetrics and Gynaecology, DMSD San Paolo, University of Milan, Milan, Italy; 9 Division of Neuropsychiatry and Brain Imaging, Department of Psychiatry (UPK), Psychiatric University Clinics Basel, University of Basel, Basel, Switzerland; 10 Department of Obstetrics and Gynecology, Sant’Orsola-Malpighi Hospital, University of Bologna, Bologna, Italy

**Keywords:** Perinatal depression, Sleep, Light therapy, Circadian rhythms, Chronobiology, Sleep disorders, Actigraphy, Polysomnography, Pregnancy

## Abstract

**Background:**

Perinatal depression (PND) has an overall estimated prevalence of roughly 12 %. Untreated PND has significant negative consequences not only on the health of the mothers, but also on the physical, emotional and cognitive development of their children. No certain risk factors are known to predict PND and no completely safe drug treatments are available during pregnancy and breastfeeding. Sleep and depression are strongly related to each other because of a solid reciprocal causal relationship. Bright light therapy (BLT) is a well-tested and safe treatment, effective in both depression and circadian/sleep disorders.

**Methods:**

In a 3-year longitudinal, observational, multicentre study, about 500 women will be recruited and followed-up from early pregnancy (10–15 gestational week) until 12 months after delivery. The primary aim of the present study is to systematically explore and characterize risk factors for PND by prospective sleep assessment (using wrist actigraphy, polysomnography and various sleep questionnaires) and bloodbased analysis of potential markers during the perinatal period (Life-ON study). Secondary aims are to explore the relationship between specific genetic polymorphisms and PND (substudy Life-ON1), to investigate the effectiveness of BLT in treating PND (substudy Life-ON2) and to test whether a short term trial of BLT during pregnancy can prevent PND (substudy Life-ON3).

**Discussion:**

The characterization of specific predictive and risk factors for PND may substantially contribute to improve preventive medical and social strategies for the affected women. The study results are expected to promote a better understanding of the relationship between sleep disorders and the development of PND and to confirm, in a large sample of women, the safety and efficacy of BLT both in prevention and treatment of PND.

**Trial registration:**

ClinicalTrials.gov NCT02664467. Registered 13 January 2016.

**Electronic supplementary material:**

The online version of this article (doi:10.1186/s12888-016-1086-0) contains supplementary material, which is available to authorized users.

## Background

Perinatal depression (PND) refers to the onset of major depression during pregnancy (antenatal depression, AD) and up to 12 months after delivery (postpartum depression, PPD). The estimated prevalence of PND is roughly 12 % [1] with a three-fold increase in incidence of depression during the postpartum period compared to nullipara [2]. Up to 80 % of women in the postpartum period report some kind of depressive symptoms, which range from a mild and transient form of sadness that is frequently termed “baby blues” to full diagnoses of major depression [3]. The Diagnostic and Statistical Manual of Mental Disorders, 4th edition text-revised (DSM-IV-TR) defined a specific subtype of major depressive disorder as post-partum depression (PPD) when the onset of the depressive episode occurs within 4 weeks after delivery [4]. Indeed, the risk of depression in puerperal women remains high through the first postpartum year [5], and in around 50 % of the cases the onset of depression occurs already during pregnancy. Based on this, the above mentioned, wider definition of perinatal depression (PND) has been adopted. The revised fifth edition of DSM (DSM-V) does not recognise this entity as a separate diagnosis, but as a specifier of major depression by using the term of “perinatal-onset or peripartum”, where a woman has to meet the criteria for major depressive episode with an onset in pregnancy or within 4 weeks of delivery [6]. An unrecognized or an untreated PND increases the risk of preterm delivery [7], smoking and substance use, shortening of breastfeeding [8], abusive behaviour toward children, suicide [9], negative effects on the relationship with the partner and cognitive problems in infants [10]. A significantly higher mean mother-infant daily cost was estimated by the British health system for women with PND compared to women without PND [11]. The absence of defined objective markers for PND diagnosis, which is purely based on the clinical interview and therefore examiner-dependent, is a significant limit to unequivocal and early recognition of the disease. Moreover, to date, except for a history of depression or positive familiar psychiatric history, no certain medical risk factors are known to predict PND [12]. With the exception of theoretical abnormalities in hypothalamic-pituitary-adrenal axis activity and possible genetic risk factors that are shared with a variety of psychiatric disorders, the etiopathogenesis of PND is still unknown. Once PND is recognized, no definitely safe pharmacologic treatments are available during pregnancy and breastfeeding [13], and the concurrent lack of preventive therapies exposes the health of women and children to the PND related risks. Placebo-controlled, randomized trials of antidepressant substances in this population are lacking, and the methodological flaws of available studies limit the generalizability of the findings. A large meta-analysis recently concluded that evidence over benefits/harms of available compounds cannot adequately support informed decisions about the treatment of PND [14]. Furthermore, teratogenicity concerns commonly influence women’s acceptance of potentially effective treatments. Therefore, a safe and effective treatment remains an urgent, currently unmet, clinical need.

The main aims of the present project are to explore the field of sleep in search of possible causative or predictive markers of PND and to test the safety and efficacy of bright light treatment (BLT) in PND. Sleep and depression are strongly related to each other with a solid reciprocal causal connection. There are several lines of evidence that suggest a pivotal role of perinatal-related sleep changes in the genesis of depression during this period. Of special importance for the present project is the consistent finding that specific sleep abnormalities are already observed in never-depressed high-risk probands and predict the subsequent onset of depression [15, 16].

BLT is a well-tested and safe therapy, which is effective in both depression [17, 18] and circadian/sleep disorders [19]. BLT is currently the treatment of choice for seasonal depression [20]. So far, the application of BLT in the treatment of PND has been limited but with overall positive results. BLT for 3 to 6 weeks improved depression and appeared to be a safe therapy for females during pregnancy and postpartum in few small open label trials [21–26].

### Sleep disorder, chronobiology and depression

A bidirectional relationship between sleep disorders and depression is well established. Affective disorders, such as major depression, are strongly associated with disruption in circadian rhythms, as demonstrated by the identification of polymorphisms in specific circadian genes that are associated with these disorders. The complex interaction between circadian genes and mood-related neurotransmitter systems, in addition to the impact of chronic stress on rhythms, are considered the mechanisms underlying this relationship [27]. Further than sleep, other circadian rhythms like hormone secretion (cortisol, prolactin, growth hormone, cortisol), body temperature and cognitive performance are impaired in patients affected by depression [28]. Sleep deprivation has been demonstrated to be the strongest therapy in the acute phase of depression [29].

Therefore, it is not surprising that the DSM-V includes changes in sleep duration (insomnia or hypersomnia) among the diagnostic criteria for major depressive disorder as an additional symptom. Mood disorders are commonly associated with subjective complaints of insomnia (difficulty falling asleep, restless sleep, early morning awakening, decrease sleep duration); sleep is less deep and refreshing and a high prevalence of disturbing dreams is reported by depressed patients.

The nocturnal recording of sleep (polysomnography - PSG) provides reliable biomarkers of depression. Objective sleep markers in depressed patients concern sleep disturbances such as prolonged sleep latency (SL), increased wake after sleep onset (WASO), increased early morning wake time, decreased total sleep time (TST), decreased sleep efficiency (SE = percentage of sleep on the total bed time), decreased slow waves sleep (SWS), decreased REM sleep latency, increased REM amount [30]. Nevertheless, mood disorders are also commonly found in patients affected by sleep disorders (i.e. insomnia [31], sleep apnoea [32], and restless legs syndrome [33]).

### Sleep disorders during pregnancy and perinatal depression

Sleep abnormalities are highly prevalent during pregnancy [34]. In the first trimester of pregnancy women often complain about fatigue and excessive daytime sleepiness. In the third trimester there is a high prevalence of insomnia, sleep related breathing disorders (i.e. obstructive sleep apnea syndrome) [35, 36], and restless legs syndrome (RLS) [37]. Several studies demonstrated a solid association between poor sleep quality, sleep disorders and post-partum mental disorders such as psychosis, anxiety and overall depression [38]. The occurrence of RLS during pregnancy is related to an increased risk of PND [39]. These findings call for a comprehensive evaluation of sleep and related disorders during pregnancy, suggesting sleep as a black box hiding possible precious candidate features for PND prediction. An early recognition of these sleep-related features during pregnancy would be highly valuable for an accompanying preventive or psychoeducational intervention in affected women, avoiding potential devastating maternal and foetal consequences.

### Bright light treatment for perinatal depression

The treatments of choice for major depression are antidepressant drugs. However, no drugs belonging to antidepressant classes (tricyclic, SSRI, SNRI) are unequivocally safe during pregnancy and breastfeeding [13]. BLT is currently the treatment of choice for seasonal affective disorders [20, 40] and also well-known as an effective treatment for non-seasonal depression [41]. Few side effects related to this treatment are reported and include jumpiness/jitteriness, nausea and headache [42]. In a cohort of untreated patients with normal ocular-retinal status, ophthalmologic evaluations did not document any acute light-induced pathology or long-term sequelae [43].

There is increasing evidence that depression is associated with misalignment of the circadian rhythm and that the well-established effects of BLT on circadian synchronization are associated with its antidepressant effects [44]. BLT promotes the resynchronization of the suprachiasmatic nucleus (SCN), the master circadian pacemaker, which is impaired in depressed patients. The firing of SCN neurons is modulated by an endogenous rhythm and by the response to photic/non-photic information received from afferent inputs, which mainly depends on the day-night cycle [44]. The effect of light on the SCN is mediated by the multisynaptic circuit of the retino-hypotalamic tract, which connects retinal photoreceptors (especially the retinal ganglion cells containing melanopsin) to the anterior hypothalamus where the SCN is located [45].

The antidepressant effect of BLT is also mediated by biogenic amines, especially serotonin. In patients successfully treated with BLT and in remission, depletion of tryptophan, the amino acid precursor of serotonin, reversed the effects of BLT on mood [46, 47]. In addition, the mood lowering effect of tryptophan depletion is blocked under bright light conditions [48] again supporting the notion that BLT affects serotonergic neurotransmission. Since alterations in tryptophan [49] and the hypothalamic pituitary adrenal (HPA) axis activity [50, 51] during pregnancy have been implicated in serotonin dysregulation and PND, this provides a possible rationale for the application of BLT in PND. As detailed above, a further rationale is the expectation that BLT’s positive effects on sleep disturbances and alertness during the perinatal period will indirectly improve depressive symptoms.

Because of its well-established antidepressant efficacy and low side-effect profile, the benefit of BLT on mood disorders during pregnancy and post-natal period has been already investigated in small pioneering open label trials [21–25] (Table 1). Overall, 3- to 6-week morning BLT for 30 to 60 min improved several depression scores by close to 50 %. Moreover, in these trials BLT appeared to be a safe treatment for females during pregnancy and puerperium. These positive results pave the way to larger randomized trials with the aim of determining the effectiveness of BLT in perinatal depression, and the safety and feasibility of this therapy [26].

**Table 1:** Published studies on the efficacy of bright light therapy in perinatal depression. Adapted from Crowley et Youngstedt [26]

Study	Subjects (n), gestational week (w)	Design, patients vs. controls (n), duration (w)	Bright light treatment	Outcomes	Adverse effects
Antenatal depression					
Oren et al. 2002 [23]	*n* = 16	OL	10,000 lux, 60 min morning (10 min after awakening)	SIGH-SAD decreased by 49 % after 3 w, by 59 % after 5 w	2 patients experienced nausea
	23 ± 7 w	3–5 w			
Epperson 2004 [22]	*n* = 10	R PC PG	7,000 lux vs. 500 lux, 60 min morning (10 min after awakening)	no difference vs. placebo, SIGH-SAD improved in both groups by 45 %	Irritable hypomania in one subject resolved after reduction of light exposure
	20 ± 8 w	5 vs. 5			
		5 w			
Wirz-Justice et al. 2011 [25]	*n* = 26	R PC DB PG	7,000 lux vs. 70 lux red light, 60 min morning (10 min after awakening)	significant greater improvement with active treatment (SIGH-HADS 58 % vs. 41 %, HDRS 64 % vs. 38 %)	No clinically meaningful side effects
	~25 w	16 vs. 10			
		5 w			
Postpartum depression					
Corral 2000 [21]	*n* = 2	OL	10,000 lux 30 min morning (7:00–9:30)	HDRS decreased by 38 and 43 %	no adverse side effects
		4 w			
Corral 2007 [24]	*n* = 15	R PC PG	10,000 lux vs. 600 lux red light, 30 min morning (7:00–9:00)	no difference vs. placebo, SIGH-SAD improved in both groups by 49 %	no adverse side effects
		10 vs. 5			
		5 w			

*Legend*: *OL* open-label, *R* randomized, *PC* placebo-controlled, *PG* parallel group, *DB* double-blind

## Methods/design

### Working hypothesis and outcomes

As already mentioned, PND is highly prevalent and is associated with maternal and foetal negative outcomes; however, its pathophysiology is still largely unknown and besides an increased risk for females with prior depression, no clear risk profile has been yet established. Sleep abnormalities have higher prevalence during pregnancy compared to the general population, but it is not clear whether they could predict the development of PND. Therefore, an increased knowledge of antecedents and risk factors will provide important information about causes, prevention, and treatment of PND. At the same time, since antidepressant drugs are not definitely safe during pregnancy and breastfeeding, there is an urgent need for safe and efficacious treatments of PND with minimal side effects. Hence, the theory behind the project is that the sleep structure during pregnancy contains essential information for the early identification of women who will develop PND. Second, given its efficacy in depression not related to pregnancy, we reason that BLT might be an easy, home-based, self-administered and safe treatment option to prevent PND in women during the early stages of pregnancy and to treat an already established PND during pregnancy and the puerperium.

### Null hypothesis

Neither subjective or polysomnographic sleep features or sleep disorders can be related to development of PND. BLT is not effective in preventing or treating PND.

### Specific aims

#### Primary aim

To systematically and prospectively explore sleep and sleep-related parameters, as well as mood changes during pregnancy and the postpartum in order to identify reliable, potential early diagnostic markers of PND (Life-ON study).

#### Secondary aims

1. To investigate the relationship between specific genetic polymorphisms and the development of depression during pregnancy and the postpartum (substudy Life-ON 1)
2. To investigate the effectiveness of BLT in treating PND (substudy Life-ON 2).
3. To test whether a short term trial by BLT in non-depressed pregnant women can prevent the onset of PND (substudy Life-ON 3)

### Description of the study

The Life-ON Project is a prospective, both observational and interventional, multicentre [three sleep centres in Northern Italy (Bologna, Milan, Turin) and one in Southern Switzerland (Lugano)], non-profit, without medicines, cohort study, which will include about 500 women, who will be longitudinally followed from early pregnancy (10–15 gestational week) until 12 months after delivery. Pregnant females attending the outpatient clinics of the involved centres and who gave voluntary written informed consent to participate in the study (Life-ON) will be included consecutively. Inclusion and exclusion criteria are listed in Table 2.

**Table 2:** Inclusion and exclusion criteria

Inclusion criteria	Exclusion criteria
• Age 18–45 years • Free of any major medical condition • Normal ocular function • Gestational age between 10 to 15 weeks at time of screening • Written informed consent	• Diagnosis of bipolar I or II disorder (DSM-5) • Recent history (less than 6 months) or current major depression or an Edinburgh Postnatal Depression Scale (EPDS) score > 12 at time of inclusion • Any psychotic episode, substance abuse, recent history of suicide attempt (less than 12 months) • Use of antidepressants or other pharmacologic treatments for depression in the last 6 months • Intrauterine fetal death

Participating women will undergo scheduled clinical examinations by a multidisciplinary team including a gynaecologist and/or obstetrician, a psychologist or psychiatrist and a neurologist expert in sleep disorders. During the periodic follow-up visits, medical/gynaecological, psychological and sleep will be assessed by structured interviews and validated scales and questionnaires (Table 3).

**Table 3:** Psychiatric and sleep assessment tools

Psychiatric assessment tools
• MINI-International Neuropsychiatric Interview (MINI), clinician rated [53] • MINI-International Neuropsychiatric Interview Plus (MINI Plus), clinician rated • Edinburgh Postnatal Depression Scale (EPDS), self-administered (depression > 12) [54] • Visual Analog Scale for depression (VAS), self-administered (ranging between 0 and 10) [55] • Hamilton Depression Rating Scale - 21 items (HDRS-21), clinician-rated (14–18 = moderate depression, 19–22 = severe depression, ≥ 23 = very severe depression) [56] • Montgomery-Asberg Depression Rating Scale (MADRS), clinician-rated (normal 0–6; mild depression 7–19; moderate depression 20–34; severe depression >34) [57] • Temperament and Character Inventory (TCI), self-administered [58] _•_ Interview for Recent Life Events (IRLE), self-administered [59]
Sleep assessment tools
• Pittsburgh Sleep Quality Index (PSQI), self-administered (good sleeper <5; poor sleeper ≥5) [60] • Insomnia Severity Index (ISI), self-administered [normal ≤7; sub threshold insomnia 8–14; clinical insomnia (moderate severity) 15–21; clinical insomnia (severe) 22–28] [61] • Epworth Sleepiness Scale (ESS), self-administered (normal <10; pathological ≥ 10) [62] • RLS interview (5 criteria), clinician rated [63] and the International RLS Study Group Rating Scale (if RLS diagnosed) [64] • Munich Parasomnia Screening (MUPS), self-administered [65] • Morningness-Eveningness Questionnaire (MEQ), self-administered (16–30 “definite evening types”, 31–41 “moderate evening types”, 42–58 “intermediate types”, 59–69 “moderate morning types”, 70–86 “definite morning types”) [66] • One night of home polysomnography (PSG) • Wrist Actigraphy (7-days of consecutive home recording)

**Fig. 1:**
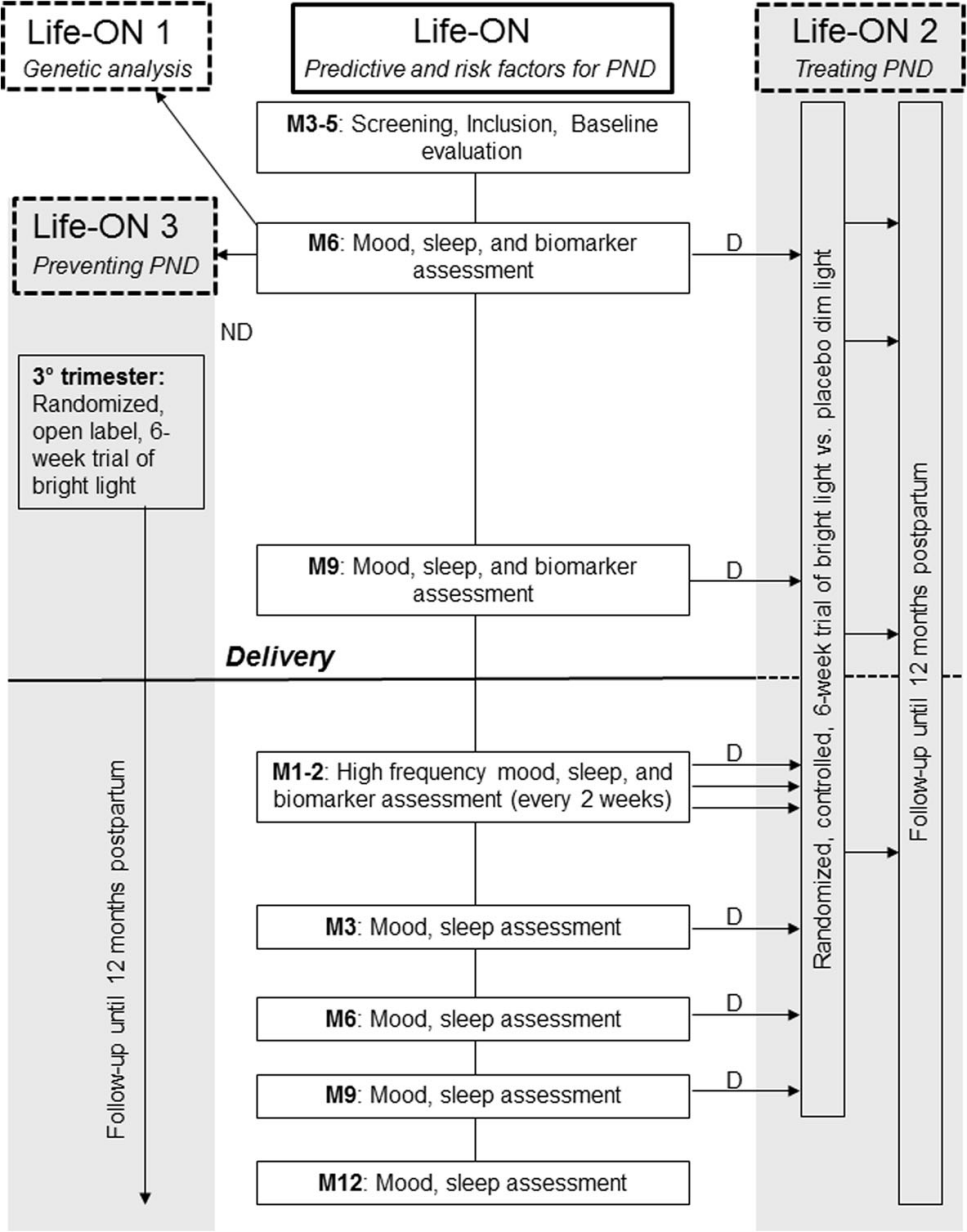
Flowchart of the study design. Legend: D: depression; M: month; ND: no depression; PND: perinatal depression; PPD: postpartum depression

Pregnant women, who will have accepted to participate in the main observational Life-ON study, will also be asked to additionally and voluntarily take in part in the Life-ON 1 substudy (genetic investigation). The substudy Life-ON 2 will be proposed to women who develop PND during pregnancy or during the 9 months’ period after delivery. A random population of non-depressed pregnant women will be asked to participate in the preventive BLT trial Life-ON 3 (substudy). A specific dedicated written informed consent is necessary to participate in each of the above mentioned substudy, and woman can withdraw their consent at any time (Fig. 1).

#### Concomitant drugs

The use of any drug prescribed in the normal clinical pathway will be allowed during the study.

### Experimental design – Life-ON study

Life-ON is an observational, longitudinal study, which will systematically and prospectively explore sleep and sleep-related parameters during pregnancy and their correlation with the onset of PND. The expected outcome is to identify reliable, potential, early diagnostic markers of PND.

Demographic and gynaecological/obstetrical data will be collected for all women included in the study. Mood, sleep quality and disorders, and other biomarkers will be assessed at regular intervals during the perinatal period, until 12 months postpartum, in all participants (Additional file 1: Table S1a).

Sleep investigations will include an interview with a sleep expert to determine lifetime and current sleep disturbances, supplemented with questionnaires assessing sleep quality, insomnia, daytime sleepiness, circadian chronotype, sleep-related movement disorders and parasomnias. Unattended ambulatory polysomnography (PSG) will be conducted in all participants between the 23th and 25th gestational week. This will include electroencephalogram (F3, C3,O1 referenced to the contralateral mastoid, M2), bilateral electro-oculogram, surface electromyogram of submentalis muscle and bilateral tibialis anterior muscles, electrocardiogram and sleep respiratory parameters (nasal air flow, thoracic and abdominal respiratory effort, oxygen saturation).

Sleep will be scored by a sleep specialist according to the international guidelines [52]. The rest/activity cycles during pregnancy and postpartum will be assessed by 7-days actigraphic recordings repeated for 3 times: between the 23th and 25th gestational week, around the 3rd (between 90 and 105 days) and between the 11th and the 12th month after delivery.

All participants will undergo a psychiatric and psychological evaluation, including semi-structured interviews and questionnaires for screening depression, stressful life events and selected personality traits. Depressive symptoms will be assessed at the time of study inclusion and approximately every 3 months until the 12th month postpartum with a higher sampling rate (every two weeks) during the two postpartum months; the diagnosis of PND will be always established using structured clinical interviews conducted by staff psychologists/psychiatrists.

### Experimental design – substudy Life-ON 1

Life-ON 1 substudy consists in a genetic test on a blood sample taken during the 23rd-25th gestational week, with the aim to identify possible specific genetic polymorphisms, that may be associated with PND. Part of the blood samples taken at inclusion will be processed and stored in a bio-bank for future use.

### Experimental design – substudy Life-ON 2

Life-ON 2 is a longitudinal and interventional trial, which will investigate the effectiveness of BLT in treating PND. Women with minor or major depression, diagnosed by a psychiatric interview or with an EPDS score >12 at any time from study inclusion to 9 months postpartum, will be invited to participate in the randomized-controlled BLT trial. Participants will be randomised on a 1:1 basis to receive 6 weeks of morning BLT or dim light placebo. The trial will be single blind. Subjects will be asked to maintain their habitual bedtime and wake-up schedule, and to begin the light treatment within 20 min of habitual wake-up time. Subjects will be instructed to sit in front of the light box (Philips Energy Up HF 3419) at a specified distance (around 30 cm) and to receive an active light dose (10’000 lux) or placebo dim light (50 lux) for ½ hour. Safety will be monitored with the self-report version of the Systematic Assessment of Treatment Emergent Effects (SAFTEE). Similarly to Life-ON, all participants will be followed up until 12 months postpartum.

### Experimental design – substudy Life-ON 3

Life-ON 3 is a longitudinal and interventional trial, in non-depressed women, which will investigate the effectiveness of BLT during pregnancy in preventing PND. A subsample of 80 subjects without depression will be randomly, consecutively recruited to receive 6-week open-label BLT during the third trimester of pregnancy. The selected participants will receive ½ hour of morning BLT (10’000 lux, Philips Energy Up HF 3419) for 6 weeks. Subjects will be asked to maintain their habitual bedtime and wake-up schedule, and BLT is planned to commence within 20 min of habitual wake-up time. Safety will be monitored with the Systematic Assessment of Treatment Emergent Effects (SAFTEE). All participants will be followed-up until 12 months postpartum and the main outcome will be the incidence of postpartum depression in subjects receiving BLT compared to subjects with no treatment.

### Safety assessment

The safety assessment will consist of monitoring and recording of Adverse Reactions (ADRs) and Serious Adverse Event (SAE) only for substudies involving treatment with BLT.

### Determination of sample size

This prospective, longitudinal cohort study will include 500 pregnant females. Given that we consecutively include participants, we judge this sample size as sufficient to generate estimates for the prevalence of perinatal depression, sleep disturbances, and sleep disorders during pregnancy and postpartum. Based on a meta-analysis of prevalence and incidence studies we expect the cumulative incidence of minor and major depression during late pregnancy and postpartum to be at least 20 % (*n* = 100). Taking into consideration that we plan to include 80 females in a trial of light treatment for the prevention of postpartum depression, we expect that approximately 300 females will be symptom free, while 100 to 150 females will experience minor or major depression during the perinatal period. For the study of risk factors involved into the development of perinatal depression, our sample size will enable the detection of small to moderate effects (d > 0.3) with an adequate statistical power (≥80 %).

For the Life-ON study no significance threshold a priori is considered.

The main outcomes of Life-ON 2 will be the change of depressive symptoms, response and remission rates after 6-week bright light treatment versus placebo. Based on a previous meta-analysis we expect that the prevalence of depression during the perinatal period will be at least 25 %, which would qualify 125 participants for inclusion in the treatment trial. From our previous experiences we anticipate an attrition rate of 25 % for reasons of refusal, non-compliance, or necessity for additional/alternative treatment. A conservative estimate would, therefore, be that 80 females will complete the treatment trial. At the same time, weekly monitoring is expected to increase compliance and reduce attrition and the available data suggests that prolongation of the treatment duration to 6 weeks will increase the bright light versus placebo difference. Our conservatively planned sample size (40 versus 40) will enable us to demonstrate medium effects (d >0.56) with a one-side error level of 0.05 and adequate statistical power (≥80 %).

Concerning the substudy Life-ON 3, previous meta-analyses have estimated an incidence of 15 to 49 % for the onset of minor and major depression during a 12 months’ postpartum period. Our sample size calculation is based on an assumed incidence of 20 % in females receiving no treatment and we expect this incidence to be reduced to 5 % in females receiving bright light treatment during late pregnancy. With a planned allocation ratio of 1:3 and a one-sided alpha level of 0.05, comparison of 43 treated females to 172 untreated subjects will yield an adequate statistical power (≥80 %). Assuming an even lower incidence of 15 % in untreated subjects, sample size calculations indicate that inclusion of 79 vs. 314 subjects will be needed to demonstrate a reduced incidence (5 %) with adequate statistical power. We therefore plan to block-randomise consecutive participants on a 1:3 basis to BTL versus no treatment until 80 participants have received active light treatment.

### Statistical analyses

Standard parametric or non-parametric statistical tests will be employed to explore differences between females with onset of depression to those remaining symptom free. To take full advantage of the longitudinal study design, generalized mixed modelling will be employed to test whether sleep-related and other fixed or variable markers can predict the onset of perinatal depression, possibly in a time-associated manner. The evaluation of BLT effectiveness for perinatal depression should take into account that this therapy will be applied at different times during the perinatal period; therefore, this will be included as a covariate in the ANCOVA (continuous data) and logistic regression analyses (dichotomous data, proportions). Regarding the efficacy of BLT in prevention of post-partum depression we will use logistic regression analysis to compare incidence rates between the group of not-depressed patients who received light therapy during pregnancy versus untreated subjects. For all analyses *p* values < 0.05 will be considered significant. The statistical analysis will be performed using a dedicated software (SPSS, IL, USA).

## Discussion

The presented project aims to address several questions concerning PND, including epidemiology, pathophysiology, prevention and treatment. It may substantially contribute to a better knowledge of the relationship between sleep disorders and PND, as well as of the feasibility, safety and efficacy of BLT both in prevention and treatment of depression during pregnancy and the postpartum period. The characterization of specific risk factors for PND will critically contribute to the development of preventive medical and social strategies for the affected women and alert the healthcare community to this relevant and frequent mental health problem. Finally, we expect that our research will significantly contribute to improve care and treatment for women during and after pregnancy and ultimately to the long-term wellbeing of mothers, children and their families.

## Additional file

Additional file 1: Table S1a Schedule of assessments. MINI: MINI International Neuropsychiatric Interview; MINI Plus: MINI International Neuropsychiatric Interview Plus; EPDS: Edinburgh Postnatal Depression Scale; VAS: Visual Analog Scale; HDRS-21: Hamilton Depression Rating Scale – 21 items (during trial also on days 0 – 21 – 42 of treatment); MADRS: Montgomery-Asberg Depression Rating Scale (during trial also on days 0 – 21 – 42 of treatment); TCI: Temperament and Character Inventory; IRLE: Interview for Recent Life Events; PSQI: Pittsburgh Sleep Quality Index; ISI: Insomnia Severity Index; ESS: Epworth Sleepiness Scale; RLS: Retless Legs Syndrome; MEQ: Morningness-Eveningness Questionnaire; PSG: Polisomnography; SAFTEE: Systematic Assessment of Treatment Emergent Effects (during trial after 3 and 7 weeks of treatment). (DOCX 30 kb)

## Abbreviations

BLT: Bright light therapy
CRF: Case report form
ECG: Electrocardiogram
EEG: Electroencephalography
EMG: Electromyography
EOG: Electrooculography
EPDS: Edinburgh perinatal depression scale
ESS: Epworth sleepiness scale
GCP: Good clinical practice
HDRS-21: Hamilton depression rating scale – 21 items
IRLE: Interview for recent life events
ISI: Insomnia severity index
MADRS: Montgomery-Asberg depression rating scale
MDD: Major depression disorder
MEQ: Morningness-eveningness questionnaire
MINI-Plus: MINI international neuropsychiatric interview - plus
MINI: MINI international neuropsychiatric interview
MUPS: Munich parasomnia screening
PLM: Periodic leg movements
PND: Perinatal depression
PSG: Polisomnography
PSQI: Pittsburgh sleep quality index
RLS: Retless legs syndrome
SAFTEE: Systematic assessment of treatment emergent effects
SWS: Slow wave sleep
TCI: Temperament and character inventory
VAS: Visual analogic scale
