# Supplementary material for: Chronobiology, sleep-related risk factors and light therapy in perinatal depression: the “Life-ON” project
Source: BMC Psychiatry. 2016 Nov 4;16:374. doi: 10.1186/s12888-016-1086-0 (PMC5225570; doi:10.1186/s12888-016-1086-0)
Supplement: Additional file 1: Table S1a — Schedule of assessments. MINI: MINI International Neuropsychiatric Interview; MINI Plus: MINI International Neuropsychiatric Interview Plus; EPDS: Edinburgh Postnatal Depression Scale; VAS: Visual Analog Scale; HDRS-21: Hamilton Depression Rating Scale – 21 items (during trial also on days 0 – 21 – 42 of treatment); MADRS: Montgomery-Asberg Depression Rating Scale (during trial also on days 0 – 21 – 42 of treatment); TCI: Temperament and Character Inventory; IRLE: Interview for Recent Life Events; PSQI: Pittsburgh Sleep Quality Index; ISI: Insomnia Severity Index; ESS: Epworth Sleepiness Scale; RLS: Retless Legs Syndrome; MEQ: Morningness-Eveningness Questionnaire; PSG: Polisomnography; SAFTEE: Systematic Assessment of Treatment Emergent Effects (during trial after 3 and 7 weeks of treatment). (DOCX 30 kb) [file 12888_2016_1086_MOESM1_ESM.docx]

| **Life-ON (Main Study)** | | | | | | | | | | | | | |
| --- | --- | --- | --- | --- | --- | --- | --- | --- | --- | --- | --- | --- | --- |
|  | ***PREPARTUM*** | | | ***POSTPARTUM*** | | | | | | | | | |
|  | V1 | V2 | V3 | V4 | V5 | | V6 | | V7 | V8 | V9 | V10 | V11 |
|  | 10-15W | 23-25W | 34-36W | 1W  (5-12gg) | 3W^T^  (19-26gg) | | 5W  (33-40 gg) | | 7W^T^  (47-54gg) | 3M  (90-105gg) | 6M  (180-195gg) | 9M^T^  (270-285gg) | 11-12M |
|  | | | | | | | | | | | | | |
| **Demographic assessment** | x |  |  |  |  | | |  |  |  |  |  |  |
|  | | | | | | | | | | | | | |
| **Gynecologic evaluation** | x |  |  | x |  | | | x |  | x | x |  | x |
|  | | | | | | | | | | | | | |
| **Psychiatric evaluation** |  |  |  |  |  | | |  |  |  |  |  |  |
| - MINI Plus | x |  |  |  |  | | |  |  |  |  |  |  |
| - MINI |  |  | x |  |  | | | x |  |  | x |  | x |
| - EPDS + VAS | x | x | x | x | x | | | x | x | x | x | x | x |
| - HDRS-21 | x |  | x |  |  | | | x |  |  | x |  | x |
| - MADRS | x |  | x |  |  | | | x |  |  | x |  | x |
| - TCI | x |  |  |  |  | | |  |  |  |  |  |  |
| - IRLE | x |  |  |  |  | | |  |  |  |  |  |  |
|  | | | | | | | | | | | | | |
| **Sleep evaluation** |  |  |  |  |  | | |  |  |  |  |  |  |
| - PSQI | x | x | x |  | x | | |  |  | x | x |  | x |
| - ISI | x | x | x |  | x | | |  | x | x | x | x | x |
| - ESS | x | x | x |  | x | | |  |  | x | x | x | x |
| - RLS criteria + severity scale | x | x | x | x |  | | | x |  | x | x | x | x |
| - Parasomnia scale | x | x | x |  |  | | |  |  | x |  |  | x |
| - MEQ | x |  |  |  |  | | |  |  |  |  |  |  |
| - Actigraphy |  | x |  |  |  | | |  |  | x |  |  | x |
| - PSG |  | x |  |  |  | | |  |  |  |  |  |  |
|  | | | | | | | | | | | | | |
| **Blood tests** (haemochrome, progesterone, estrogens, prolactin, TSH, iron, magnesium, ferritin, transferrin, vitamine B12, folic acid, creatinine) | x | x | x |  |  | | | x |  |  |  |  |  |
| **Life-ON 1 (Genetic Substudy)** | | | | | | | | | | | | | |
|  | ***PREPARTUM*** | | | ***POSTPARTUM*** | | | | | | | | | |
|  | V1 | V2 | V3 | V4 | V5 | | | V6 | V7 | V8 | V9 | V10 | V11 |
|  | 10-15W | 23-25W | 34-36W | 1W  (5-12gg) | 3W^T^  (19-26gg) | | | 5W  (33-40 gg) | 7W^T^  (47-54gg) | 3M  (90-05gg) | 6M  (180-195gg) | 9M^T^  (270-285gg) | 11-12M |
| **Genetic tests** (blood sample) |  | x |  |  |  | | |  |  |  |  |  |  |
| **Life-ON 2 (BLT Therapeutic Substudy)** | | | | | | | | | | | | | |
|  | ***PREPARTUM*** | | | ***POSTPARTUM*** | | | | | | | | | |
|  | V1 | V2 | V3 | V4 | V5 | | V6 | | V7 | V8 | V9 | V10 | V11 |
|  | 10-15W | 23-25W | 34-36W | 1W  (5-12gg) | 3W^T^  (19-26gg) | | 5W  (33-40 gg) | | 7W^T^  (47-54gg) | 3M  (90-05gg) | 6M  (180-195gg) | 9M^T^  (270-285gg) | 11-12M |
|  | | | | | | | | | | | | | |
| **Demographic assessment** |  |  |  |  |  |  | | |  |  |  |  |  |
|  | | | | | | | | | | | | | |
| **Gynecologic evaluation** |  | x | x | x |  | x | | |  | x | x |  | x |
|  | | | | | | | | | | | | | |
| **Psichiatric evaluation** |  |  |  |  |  |  | | |  |  |  |  |  |
| - MINI Plus |  |  |  |  |  |  | | |  |  |  |  |  |
| - MINI |  |  |  |  |  |  | | |  | x | x | x | x |
| - EPDS + VAS |  | x | x | x | x | x | | | x | x | x | x | x |
| - HDRS-21* |  |  |  |  |  |  | | |  | x | x | x | x |
| - MADRS* |  |  |  |  |  |  | | |  | x | x | x | x |
| - TCI |  |  |  |  |  |  | | |  |  |  |  |  |
| - IRLE |  |  |  |  |  |  | | |  |  |  |  |  |
| *during trial also on day 0-21-42 of treatment | | | | | | | | | | | | | |
|  | | | | | | | | | | | | | |
| **Sleep evaluation** |  |  |  |  |  |  | | |  |  |  |  |  |
| - PSQI |  | x | x |  | x |  | | |  | x | x |  | x |
| - ISI |  | x | x |  | x |  | | | x | x | x | x | x |
| - ESS |  | x | x |  | x |  | | |  | x | x | x | x |
| - RLS criteria + severity scale |  | x | x | x |  | x | | |  | x | x | x | x |
| - Parasomnia scale |  | x | x |  |  |  | | |  | x |  |  | x |
| - MEQ |  |  |  |  |  |  | | |  |  |  |  |  |
| - Actigraphy |  | x |  |  |  |  | | |  | x |  |  | x |
| - PSG |  | x |  |  |  |  | | |  |  |  |  |  |
|  | | | | | | | | | | | | | |
| **SAFTEE§** |  |  | x | x | x | x | | | x | x | x | x | x |
| §during trial after 3 and 7 weeks of treatment | | | | | | | | | | | | | |
|  | | | | | | | | | | | | | |
| **Blood test** (haemochrome, progesterone, estrogens, prolactin, TSH, iron, magnesium, ferritin, transferrin, vitamine B12, folic acid, creatinine) |  | x | x |  |  | x | | |  |  |  |  |  |
| **Life-ON 3 (BLT Preventive Substudy)** | | | | | | | | | | | | | |
|  | ***PREPARTUM*** | | | ***POSTPARTUM*** | | | | | | | | | |
|  | V1 | V2 | V3 | V4 | V5 | | V6 | | V7 | V8 | V9 | V10 | V11 |
|  | 10-15W | 23-25W | 34-36W | 1W  (5-12gg) | 3W^T^  (19-26gg) | | 5W  (33-40 gg) | | 7W^T^  (47-54gg) | 3M  (90-05gg) | 6M  (180-195gg) | 9M^T^  (270-285gg) | 11-12M |
|  | | | | | | | | | | | | | |
| **Demographic assessment** |  |  |  |  |  |  | | |  |  |  |  |  |
|  | | | | | | | | | | | | | |
| **Gynecologic evaluation** |  | x | x | x |  | x | | |  | x | x |  | x |
|  | | | | | | | | | | | | | |
| **Psichiatric evaluation** |  |  |  |  |  |  | | |  |  |  |  |  |
| - MINI Plus |  |  |  |  |  |  | | |  |  |  |  |  |
| - MINI |  |  | x |  |  | x | | |  |  | x |  | x |
| - EPDS + VAS |  | x | x | x | x | x | | | x | x | x | x | x |
| - HDRS-21* |  |  |  |  |  | x | | |  |  | x |  |  |
| - MADRS* |  |  |  |  |  | x | | |  |  | x |  | x |
| - TCI |  |  |  |  |  |  | | |  |  |  |  |  |
| - IRLE |  |  |  |  |  |  | | |  |  |  |  |  |
| *during trial also on day 0-21-42 of trreatment | | | | | | | | | | | | | |
|  | | | | | | | | | | | | | |
| **Sleep evaluation** |  |  |  |  |  |  | | |  |  |  |  |  |
| - PSQI |  | x | x |  | x |  | | |  | x | x |  | x |
| - ISI |  | x | x |  | x |  | | | x | x | x | x | x |
| - ESS |  | x | x |  | x |  | | |  | x | x | x | x |
| - RLS criteria + severity scale |  | x | x | x |  | x | | |  | x | x | x | x |
| - Parasomnia scale |  | x | x |  |  |  | | |  | x |  |  | x |
| - MEQ |  |  |  |  |  |  | | |  |  |  |  |  |
| - Actigraphy |  | x |  |  |  |  | | |  | x |  |  | x |
| - PSG |  | x |  |  |  |  | | |  |  |  |  |  |
|  |  |  |  |  |  |  | | |  |  |  |  |  |
| **SAFTEE§** |  |  | x | x | x | x | | | x | x | x | x | x |
| §during trial after 3 and 7 weeks of treatment | | | | | | | | | | | | | |
|  | | | | | | | | | | | | | |
| **Blood tests** (haemochrome, progesterone, estrogens, prolactin, TSH, iron, magnesium, ferritin, transferrin, vitamine B12, folic acid, creatinine) |  | x | x |  |  | x | | |  |  |  |  |  |
